# Space flight and central nervous system: Friends or enemies? Challenges and opportunities for neuroscience and neuro‐oncology

**DOI:** 10.1002/jnr.25066

**Published:** 2022-06-09

**Authors:** Giovanni Marfia, Stefania Elena Navone, Laura Guarnaccia, Rolando Campanella, Marco Locatelli, Monica Miozzo, Pietro Perelli, Giulio Della Morte, Leonardo Catamo, Pietro Tondo, Carmelo Campanella, Marco Lucertini, Giuseppe Ciniglio Appiani, Angelo Landolfi, Emanuele Garzia

**Affiliations:** ^1^ Laboratory of Experimental Neurosurgery and Cell Therapy, Neurosurgery Unit Fondazione IRCCS Ca' Granda Ospedale Maggiore Policlinico Milan Italy; ^2^ Aldo Ravelli' Research Center Milan Italy; ^3^ Clinical Pathology Unit Istituto di Medicina Aerospaziale “A. Mosso”, Aeronautica Militare Milan Italy; ^4^ Department of Clinical Sciences and Community Health University of Milan Milan Italy; ^5^ Department of Medical‐Surgical Physiopathology and Transplantation University of Milan Milan Italy; ^6^ Department of Health Sciences Università degli Studi di Milano Milan Italy; ^7^ Unit of Medical Genetics ASST Santi Paolo e Carlo Milan Italy; ^8^ Istituto di Medicina Aerospaziale “Aldo Di Loreto” Aeronautica Militare Rome Italy; ^9^ Italian Air Force Logistic Command Rome Italy; ^10^ Istituto di Medicina Aerospaziale “A. Mosso” Aeronautica Militare Milan Italy; ^11^ Istituto di Medicina Aerospaziale “Aldo Di Loreto” Aeronautica Militare Roma Italy

**Keywords:** aerospace medicine, brain tumors, central nervous system, microgravity, neuroscience

## Abstract

Space environment provides many challenges to pilots, astronauts, and space scientists, which are constantly subjected to unique conditions, including microgravity, radiations, hypoxic condition, absence of the day and night cycle, etc. These stressful stimuli have the potential to affect many human physiological systems, triggering physical and biological adaptive changes to re‐establish the homeostatic state. A particular concern regards the risks for the effects of spaceflight on the central nervous system (CNS), as several lines of evidence reported a great impact on neuroplasticity, cognitive functions, neurovestibular system, short‐term memory, cephalic fluid shift, reduction in motor function, and psychological disturbances, especially during long‐term missions. Aside these potential detrimental effects, the other side of the coin reflects the potential benefit of applicating space‐related conditions on Earth‐based life sciences, as cancer research. Here, we focused on examining the effect of real and simulated microgravity on CNS functions, both in humans and in cellular models, browsing the different techniques to experience or mime microgravity on‐ground. Increasing evidence demonstrate that cancer cells, and brain cancer cells in particular, are negatively affected by microgravity, in terms of alteration in cell morphology, proliferation, invasion, migration, and apoptosis, representing an advancing novel side of space‐based investigations. Overall, deeper understandings about the mechanisms by which space environment influences CNS and tumor biology may be promisingly translated into many clinical fields, ranging from aerospace medicine to neuroscience and oncology, representing an enormous pool of knowledge for the implementation of countermeasures and therapeutic applications.

**Significance**

High‐performance pilots, astronauts, and space scientists are regularly subjected to highly dynamic environmental conditions, known as “exposome,” which may potentially affect human health. Microgravity showed to importantly impact CNS, in terms of cognitive skills and neuroplasticity. Aerospace medicine and brain research should consider the urgency to evaluate short‐ and long‐term effects of microgravity on CNS, to adopt and optimize precise countermeasures to overcome pathological conditions. Further, the effect of microgravity may advance a novel tactic to deeper understand molecular mechanisms underlying neurodegenerative and neurooncological disease, with the aim of reclassifying disease risks and identifying predictive/diagnostic biomarkers and new actionable targets.

## 1 INTRODUCTION

In the course of its evolution, humanity has constantly sought to broaden its horizons, out of a thirst for knowledge, new resources, and abilities. This continuous research has led to the enormous progress of sciences and technologies, which today are at the zenith of their expansion. Thanks to the development of space flight, the intrinsic curiosity for the unknown has led mankind to explore also the universe outside our planet, discovering planets and galaxies, advancing also the concept of space tourism. One of the most relevant concern regarding space flight is the short‐ and long‐term impact of space environment on human health.

Despite the advancement of technologies and the efforts of highly qualified engineers to preserve safety and performance of astronauts, the effects of space flights and journeys on astronaut's health remain a serious concern. Aerospace pilots represent a population often working in extreme conditions, being continuously exposed to stressful stimuli which trigger physical and biological adaptive changes to reestablish the homeostatic state. The set of environmental conditions to which air and space travelers are subjected is known as “exposome.” This “space exposome” is a complex and dynamic framework that reflects the interaction of all the environmental impacts on the human body and, when combined with individual traits, can profile the outcomes of space travel on the human system (Crucian et al., 2018). To this regard, aerospace medicine is born to analyze and monitor the clinical, biological, genetic, and psychological effects of space travel on human health. Of relevance, the novel frontiers of aerospace medicine are moving toward the emerging field of personalized medicine, based on individual traits as person's genes, proteins, and metabolites related to nutrition, diet, lifestyle, and environment, to the aim of integrating information from multiple sources.

Space environment had been demonstrated to affect almost all systems of human physiology. The major health hazards of spaceflight include higher levels of damaging radiations, altered gravity fields, hypoxic condition, absence of the day and night cycle, vibration, acceleration, long periods of isolation, and confinement, together with the stress deriving from a closed and potentially hostile living place. Each of these threats is associated with its own set of physiological and performance risks, comprising alteration in the cardiovascular functions, fluid redistribution, metabolism, and immune system; muscle disorders; and motion sickness. This widely reported evidence hampered the importance to estimate the magnitude of space impact on human tissues and cellular models. For example, numerous epidemiological studies analyzing humans exposed to radiation reported an association between radiation exposure and the development of cancer and other non‐cancer health (Boice et al., 2018; Kamiya et al., 2015; Ozasa et al., 2019). The risk of radiation‐induced carcinogenesis is considered a “red” risk for exploration missions due to the high potential for detrimental impact on both quality of life and disease‐free survival post flight (Patel et al., 2020). Similarly, it has been widely observed that long‐term exposure to microgravity causes a few physiological and biochemical changes in humans as atrophy of antigravity muscles, fluid shifts, and decreased plasma volume; negative calcium balance resulting in the loss of bone; and cardiovascular deconditioning leading to orthostatic intolerance (Wolfe & Rummel, 1992).

However, several studies reported the influence of real and simulated microgravity on cell migration, proliferation, and apoptosis, both on differentiated and stem cell (Grimm et al., 2020). These mechanisms are strictly involved in many pathogenic conditions as neurodegenerative diseases, ischemia, autoimmune disorders, and tumor onset and progression (Prasad et al., 2020), suggesting the potential of microgravity as a therapeutic approach.

Relatively to microgravity impact on CNS, brain tumors deserve a particular attention, as previous groups have shown that microgravity is able to inhibit tumor progression and increase the chemosensitivity to chemotherapy of malignant gliomas, suggesting that not only detrimental effects of microgravity should be considered. Indeed, gliomas represent the most common and aggressive brain tumors, with a high mortality rate and an average survive duration less than 15 months, thus representing an extreme therapeutic challenge.

Notably, since space studies are limited by several logistic, financial, and practical restrictions, ground‐based analogues have been developed to overcome some of these problems.

Here, we provide an overview of the current knowledge of the effect of microgravity on CNS, both in human and cellular models, based on actual spaceflight and parabolic flight studies and on ground‐based techniques suitable for microgravity‐based research. Further, in this review, we explore the influence of microgravity on tumor cell biology, focusing on brain tumor cells, with the purpose of affording a new approach to the study of cancer and relative innovative therapeutic strategies.

## 2 THE BROAD SPECTRUM OF SPACE ENVIRONMENT

Space is an extreme environment for human organism. Especially during long‐term missions, astronauts experience stressful stimuli as radiation, microgravity, hypobaric environment, and acceleration forces, which in turn cause pathophysiological adaptive changes comparable to many diseases and aging process. Human body reactions induced by the extreme space conditions may improve our knowledges about the limits of the human organisms and may potentially reveal prognostic symptoms of diseases (Demontis et al., 2017). The clinical experience with radiation exposure, as radiotherapy to control the growth of primary and secondary brain tumors, proved the existence of radiation‐induced weakening effects on cognition, memory, learning, attention, executive functions, as well as behavioral changes, depression, and anxiety. Experimental data reported a correlation between radiation‐induced impairments with a structural complexity reduction of neurons, neuroinflammation, and microvascular disruption. Similarly, astronauts exposed to low doses of protons are at risk for CNS damages. In a mouse model of whole‐body proton irradiation, it has been reported that mice subjected to 0.1 to 1 Gy showed a dose‐dependent reduction of dendritic complexity and a significant decrease of dendritic spine number and density along hippocampal neurons (Parihar et al., 2015). Analogously, recent evidence arising from rodents exposed to cosmic radiation reported behavioral decrements associated with synaptic integrity and neuronal structure. In this model, irradiation caused a decrease in spine density and dendritic complexity and altered morphology of cortical neurons which mediate neurotransmission. This in turn coincided with the impairment of spatial and recognition memory, deficits in executive functions, reduced fear, and increased anxiety (Parihar et al., 2016). In a retrospective study, neuroradiological images and balance data from 27 astronauts were analyzed to determine human brain structural changes after spaceflight. Results demonstrated a volumetric decrease of gray matter, especially in the area covering the frontal and temporal poles. Increases of focal gray matter were observed within the medial primary somatosensory and motor cortex. Notably, this outcome was greater on ISS versus shuttle crew members, suggesting a space duration effect and a cumulative effect (Koppelmans et al., 2016). However, these results may be considered speculative due to the retrospective and heterogenic nature of data, thus prospective studies will be fundamental to refine structural brain changes after spaceflight in human subjects.

Interestingly, at cellular level, a recent report reveals that radiation may be responsible for neuroepigenetic changes which lead to altered gene expression, impacting learning and memory. Indeed, in mice exposed to cosmic radiation, it was observed an increase of 5‐methylcytosine (5mC) and 5‐hydroxymethylcytosine (5hmC) in the hippocampus, which coincides with increased level of DNA‐methylating enzymes DNMT3a, TET1, and TET3. This alteration was found to be associated with persistent impairments in hippocampal and cortical memory. Intriguingly, by inhibiting DNA methylation, the adverse effects of irradiation were reversed, restoring mice cognition and behavioral performance (Acharya et al., 2017). Another evidence arose from the activation of rat microglia and the increased levels of the postsynaptic density protein (PSD‐95) after cosmic radiation exposure. Activated microglia is able to regulate structural plasticity by shortening dendritic spines and arbors, whereas alterations in PSD‐95 can affect synaptic integrity by unsettling the distribution and composition of proteins and receptors in the synaptic cleft (Keith & El‐Husseini, 2008; Preissmann et al., 2012; Wake et al., 2013).

Regarding microgravity, on Earth, gravity, or *G‐force*, determines nearly all physical, chemical, and biological phenomena occurring on our planet. Everything on Earth is subjected to gravity and the weight of a person corresponds to the force exerted upon the mass of the human body by the Earth's gravitational field. The effect of gravity on an object can be completely canceled out when it experiences “free fall,” as it occurs in orbit. This state is due to microgravity, which refers to an environment in which gravity is less than that faced on Earth's surface, and it is commonly called weightlessness. The physical and biological adaptive changes occurring during space missions highlight the importance of gravity during human evolution, and an association between microgravity, aging, and disease onset. In space, this lack of normal gravity results in the loss of mechanical stimulation of cells and tissues and is therefore responsible for many of the physiological problems that astronauts experience, including bone loss (Nabavi et al., 2011; Smith & Heer, 2002), muscle loss (Bajotto & Shimomura, 2006; Fitts et al., 2010), loss of cardiovascular capacity (Convertino, 2005; Cooke & Convertino, 2005), possible defects in wound (Martinez et al., 2007), bone fracture healing (Kirchen et al., 1995), and impaired immune function (Crucian et al., 2011; Stowe et al., 2011). At CNS level, microgravity is believed to affect the brain via multiple mechanisms, including neuroplasticity alterations, vestibular deprivation, weightlessness, and cephalic fluid shift (De la Torre, 2014). It has been shown also that microgravity can alter important properties of cells including cell morphology, proliferation, and migration (Bradbury et al., 2020; Crawford‐Young, 2006). Gravitational biology and its effect on brain plasticity and cancer cell biology are of great interest and remain a current topic in space research. Therefore, from this point, we will focus on microgravity impact on CNS, both in benign and malignant cells, addressing the suitable techniques for microgravity‐based research.

## 3 SPACEFLIGHT ANALOGUES AND GROUND‐BASED TECHNIQUES TO INVESTIGATE MICROGRAVITY

Research in microgravity is indispensable to disclose the impact of gravity on biological processes and organisms. Spaceflights to the International Space Station (ISS) provide unique conditions to investigate microgravity. However, research in the near‐Earth orbit is severely constrained by the limited number of flight opportunities, as experiments should be performed autonomously, the design is reasonably difficult, the cost are highest compared to the other flight options, and the preparation requires years (Prasad et al., 2020).

For brief periods, real microgravity can be experienced by airtight capsule dropped down in an evacuated tube within a tower, like the drop tower in Bremen, Germany (Eigenbrod, 2011), an European unique facility for experiments under conditions of high‐quality weightlessness with residual gravitational accelerations in the microgravity regime. The period of maximum 4.74 s of each freely falling experiment at the Drop Tower Bremen is only limited by the height of the drop tower vacuum tube, which is fully manufactured of steal and enclosed by an outer concrete shell. In this case, the pure free fall height of each microgravity drop experiment is approximately 110 m (Eigenbrod, 2011). Notably, the capsule can also be shot from the bottom of the tube upwards, from where it falls back, effectively doubling the microgravity time, but subjecting the experiment to another high‐acceleration event (Von Kampen et al., 2006).

Parabolic flights are used for longer microgravity exposure. The airplane climbs at an increasingly steeper angle until 50° and the propulsion is reduced to purely compensate for drag. Following the peak, the airplane enters a nosedive, which ends at around a −42° decline, after which is again pulled up to a horizontal flight path. By this way, the microgravity lasts for about 22 s and is edged by two hypergravity phases with about 2 G for 20 s each, after which the airplane climbs at an increasingly steeper angle until 50° and the propulsion is reduced to purely compensate for drag (Acharya et al., 2019). Further, even longer microgravity exposure may be reached by space trip with a suborbital flight with a sounding rocket, allowing for 6 min (TEXUS) or 13 min (MAXUS/MAPHEUS) of microgravity (Sabbatini, 2014).

**FIGURE 1:**
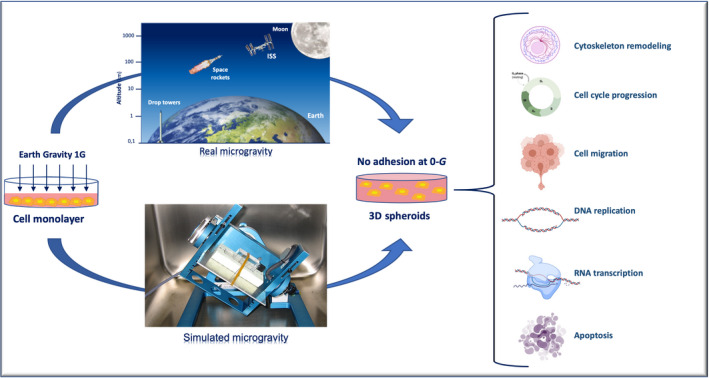
Current research platforms to conduct study with real μg in space environment. On the Earth, at 1G gravity condition, cells grow as an adhesive monolayer, whereas in orbit, with the decrease of gravity, cells lose their adhesive property, adopting a floating aspect. The random positioning machine (RPM) is a ground‐based device to simulate microgravity, in order to setup spheroid, organoid, and 3D growth cultures to investigate the effect of microgravity on cell behavior, cytoscheleton remodeling, cell cycle progression, cell migration, DNA replication, RNA transcription, and apoptosis.

On the Earth, ground‐based techniques to investigate microgravity impact on human body comprise the “Head‐down bed rest” (HDBR) method, in which the subject lies on a bed with the head tilted down by 6° (Messerotti Benvenuti et al., 2011). This condition can be engaged for short‐term investigations (e.g., 72 hr) (Liao et al., 2012), or long‐term studies (e.g., 90 days) (Roberts et al., 2010), mimicking many effects of spaceflight on the human body, such as a decrease in bone density, muscle mass, and strength and cephalic fluid shift. In parallel, the most reliable device actually available to test simulated microgravity on cellular models in vitro is the 3D‐clinostat, also called *random positioning machine* (RPM). The 3D‐clinostat is a multidirectional G‐force generator, consisting of a central platform in which a cell culture flask containing a cell monolayer is fixed, interconnected with two perpendicular arms that rotate independently of each other, thus providing continuous rotation with two axes (Becker & Souza, 2013). By this way, the 3D‐clinostat annuls the gravity vectors at the center of device, enabling cell within to experience a microgravity environment with an average of 10^−3^ G over time, thanks to which it is possible to observe a lack of sedimentation and the growth of 3D multicellular spheroids (Figure 1). The continuous rotation of the RPM provides constant randomization of the gravity vector, making this device a useful adjunct to prepare for spaceflight studies. A specialized form of clinostat is the rotating wall vessel (RWV) bioreactor (Goodwin et al., 1993; Schwarz et al., 1992), developed by NASA, which consists in a horizontally rotating vessel with no internal mechanical agitator, in which the vessel provides an environment characterized by low turbulence and shear.

The RPM has been widely used to develop culture models of malignant glioma (Takeda et al., 2009), thyroid carcinoma (Grosse et al., 2012), and leukemic cells (Cuccarolo et al., 2010), the key findings from which are discussed below.

## 4 MICROGRAVITY IMPACT ON CNS

### 4.1 Review on human studies

One of the most interestingly and scientifically sound experiment investigating how space environment affect human physiology is represented by “The NASA Twins Study,” an integrated description of the effects of a 340‐day mission on‐board the ISS.

This multidimensional analysis reported the effects of long‐term space mission on two identical twin astronauts monitored before, during, and after a 1‐year mission on‐board the ISS. Longitudinal assessments identified spaceflight‐specific changes, including altered ocular structure, cognitive decline postflight, gastrointestinal microbiota alterations, carotid artery distension, decreased body mass, genome instability, telomere elongation, transcriptional and metabolic changes, and DNA methylation changes in immune and oxidative stress‐related pathways. Some of these alterations were further affected by the stress associated with return on Earth. Intriguingly, some changes persisted even after 6 months on Earth, as some gene's expression levels, increased DNA damage, increased numbers of short telomeres, and weakened cognitive function. This behavioral, physiological, molecular, and multiomic study provide a valuable roadmap of the recognized health risks for human spaceflight, suggesting the need to optimize the planning of future long‐term space missions with tailored countermeasures (Garrett‐Bakelman et al., 2019).

At CNS level, microgravity seems to induce alterations in multiple structures and functions. For example, neurovestibular system is particularly impacted, as the otoliths that detect linear acceleration are brusquely deprived of the sense of gravity. This event affects vestibular nuclei and cortical projection, where different sensory inputs are integrated (Morita et al., 2016).

Current spaceflight studies using electroencephalography (EEG) to monitor electrocortical activity of four cosmonauts with previous experience of space flights reported an increase in the alpha rhythm in the parieto‐occipital and sensorimotor area, probably associated with gravity loss (Cheron et al., 2006). More recently, on astronauts in free‐floating aboard the International Space Station, before the space flight and afterward, it has been reported that microgravity decreased alpha rhythm of the cerebellum, the vestibular system, and the bilateral motor cortex (Cebolla et al., 2016). These observations highlight the increased request to integrate incongruent vestibular information, as well as the increased processing crucial for postural stabilization (Cebolla et al., 2016). In addition, a dry immersion study confirmed the decrease of alpha power and reported a modest increase in theta power (Kuznetsova et al., 2015). In parallel, parabolic flight studies reported a decrease of beta power, which may reflect an emotional reaction to the absence of gravity, the baroreceptor stimulation, or the lower excitation levels (Lipnicki, 2009; Schneider et al., 2008). Of relevance, HBDR studies observed contrasting results, consisting in increased alpha, beta, and theta power. These data may be explained by the fact that HDBR preserves the gravitational stimulus, thus it might not be the best model to investigate the effect of microgravity on the human brain. Furthermore, if alteration in electrocortical activity after parabolic and spaceflight reflect increased emotional stress levels, dry immersion and HDBR are associated with sense of immobilization and monotony (Marušič et al., 2014).

Also, magnetic resonance imaging (MRI) resulted effective in detecting in vivo structural, metabolic, functional, and vascular alterations induced by microgravity. A recent case study reported significant alterations in human brain functions after long‐duration spaceflight, as well as differences in the functional connectivity between cerebellum and motor cortex, and alterations in the supplementary motor area (Demertzi et al., 2016). Another observation regards the increase of intracranial pressure (ICP), measured by combining changes in intracranial volumetric parameters, pituitary morphologic structure, and aqueductal cerebrospinal fluid (CSF) hydrodynamics. The comparison of these parameters pre‐ and post‐long‐duration spaceflight determined that spaceflight was associated with increased pituitary deformation, augmented CSF hydrodynamics, and expansion of summated brain and CSF volumes. It has been recently reported that summated brain and CSF volumetric expansion persisted up to 1 year into recovery, suggesting potential permanent alterations (Kramer et al., 2020). Notably, in this context, NASA in 2010 termed this increase of ICP as Visual Impairment Intracranial Pressure Syndrome, but in 2017 it was changed to Spaceflight Associated Neuro‐Ocular Syndrome (SANS), as increased ICP was not considered to be the primary issue (Lee et al., 2017).

Contrastingly, Lawley et al. reported that prolonged periods of simulated microgravity did not cause progressive elevations in ICP, advancing the idea that the human brain may be protected by the daily circadian cycles, without which pathology may occur (Lawley et al., 2017).

In the investigation of microgravity impact on CNS, neuroplasticity deserves a particular attention.

Neuroplasticity concerns the ability of the brain to organize its structure and related functions in response to stressful stimuli or environment. Neuroplasticity can act at several levels, from synaptic plasticity at cellular level, to neural network level. MRI, EEG with evoked potentials (ERPs), and transcranial magnetic stimulation (TMS) are widely used to study neuroplasticity, in terms of cortical dynamics, changes in activity patterns, map size and cortical excitability, and task‐related or resting‐state neural activity (Van Ombergen et al., 2017). HDBR ground studies reported that microgravity inhibits cortical activity, by increasing the amplitude of delta and theta EEG low‐frequency rhythms (Vaitl et al., 1996). Notably, cortical plasticity is strictly related to learning, which represent a critical cognitive function in long‐term space missions. For example, a startle reflex habituation study has been conducted to compare subjects submitted to HDBR with sitting controls, to measure learning and brain plasticity injury (Messerotti Benvenuti et al., 2011). Being consistent with previous study reporting an impairment in astronauts' sensory and motor and complex cognitive activity (Manzey & Lorenz, 1998), the study demonstrated a microgravity‐induced lack of startle reflex plasticity in HDBR subjects, suggesting that further knowledge of learning and reasoning in the space environment is mandatory for the success of future long‐term space missions (Messerotti Benvenuti et al., 2011).

Other studies reported that prolonged HDBR method had a detrimental effect on individual executive functioning, emotion, and physiological activity (Liu et al., 2012), as well as on postural instability (Muir et al., 2011) and impaired functional mobility (Reschke et al., 2009).

Roberts et al. observed brain tissue expansion in the central frontoparietal regions and contraction in orbitofrontal regions in subjects in HDBR state and, in parallel, Liao et al. reported a decrease in gray matter in frontal brain regions and small gray matter increases in posterior parietal regions in 18 subjects (Liao et al., 2012; Roberts et al., 2010). They reported a HDBR‐induced reduction of cortical activity in the motor areas with leg representation and a decrease in corticospinal excitability by means of TMS. Interestingly, they observed that the larger the increase in motor cortex excitability, the smaller was the functional mobility impairment. A few years later, Liao et al. demonstrated also a decreased thalamic connectivity during resting‐state after 72 hr of HDBR (Liao et al., 2012). More recently, Pechenkova et al. investigated by functional MRI alterations in functional brain connectivity in cosmonauts after a long‐duration spaceflight. The authors reported changes in functional brain connectivity specific to a plantar stimulation activity occurred. The modifications observed resulted in an increased connectivity between the right and the left insulae and between the part of the right posterior supramarginal gyrus with the rest of the brain. In contrast, a decreased connectivity was reported between the right inferior parietal cortex, the vestibular nuclei and cerebellum with areas associated with motor, visual, vestibular, and proprioception functions, as well as decreased connectivity. Although the results cannot be attributed only to exposure to microgravity, because these effects are contaminated by the readjustment to earth gravity that occurred in the period between the landing and the post‐flight fMRI, the results may be really important for future research investigating neural plasticity adaptation related to microgravity (Pechenkova et al., 2019). The evidence reported suggest that space travelers face up several challenges as radiation and microgravity, which may potentially act synergistically to alter cognition, learning, and memory.

### 4.2 Review on non‐cancer cells in vivo and cellular studies

An interesting field of research concerns the effects of microgravity on human brain nervous tissue. G‐force settles the spatial relationship between cellular organelles and cytoskeletal structure, thus affecting the biochemical pathways. Accordingly, at cellular level, gravity impacts cytoarchitectural biology, influencing DNA replication, RNA transcription, protein coding, transport, and metabolism. In the CNS, oligodendrocytes are the glial cells that synthesize and maintain myelin. Oligodendrocytes guarantee the neural transmission by the formation of compact myelin segments coiling axons, which allow the saltatory conduction of electric impulse. It has been reported that oligodendrocytes, subjected to simulated microgravity, increased mitochondrial respiration and glycolysis, as well as the synthesis and secretion of fatty acids and complex lipids (Espinosa‐Jeffrey et al., 2016).

Microgravity has proved also to be able to enhance differentiation of mesenchymal stem cells into neurons, suggesting a potential new strategy for CNS degenerative diseases. Indeed, rat mesenchymal stem cells cultured with a neuronal differential medium and in a clinostat simulating microgravity expressed higher microtubule‐associated protein‐2 (MAP‐2), tyrosine hydroxylase (TH), and choline acetyltransferase (CHAT), compared to those cultured in normal gravity. Further, these cells secrete a higher amount of neurotrophines like brain‐derived growth factor (BDNF) and nerve growth factor (NGF) (Chen et al., 2011). Interestingly, also the activity of bone marrow stromal cells (BMSCs), which have attracted attention for the treatment of CNS diseases, is influenced by gravity. For example, a study conducted on mice‐derived BMSCs cultured in neural differentiation medium reported that neural‐induced BMSCs cultured at 1 G exhibited neural differentiation, unlike those cultured in microgravity condition. Further, after intravenous injection in a mouse model of cerebral contusion, BMSCs cultured under simulated microgravity showed greater survival in the injured region, expressing increased level of CXCR4 on cell membrane. Also, the motor function of the grafted mice improved significantly (Yuge et al., 2011).

A potential problem with microgravity exposure is related to oxidative stress. In the hippocampus, for example, oxidative mechanisms are closely associated with the enhanced activation of glucocorticoid receptors during stress response, as microgravity. An interesting study was conducted by Sarkar et al. in mice subjected to simulated by microgravity obtained by suspending by their tail in the center of the cage but allowing to touch the floor with their front paws and left for 7 days. Data resulting reported that mice hippocampi decrease the levels of β‐synuclein and pyruvate dehydrogenase (PDK‐1) (Sarkar et al., 2008). β‐Synuclein is a molecular chaperone, known for preventing the aggregation of nonconforming proteins. The decreased level of β‐synunclein, found under microgravity state, may lead to the increased incidence of anomalous protein aggregations. On the other hand, PDK‐1 plays a key role in the regulation of glucose and fatty acid metabolism and homeostasis, influencing cellular responses to hypoxia and oxidative stress by protecting cells against apoptosis. A recent study assessed the long‐term transcriptional effects of spaceflight analog conditions in a mouse model, by simulating microgravity via hindlimb unloading (HLU) and/or low‐dose γ‐ray irradiation (LDR). The results demonstrated a minimal alteration in gene expression and cytosine methylation after a single HLU or LDR treatment. On the contrary, a combination of HLU and LDR produced multiple alterations in gene expression and promoter methylation in pathway involved in neurogenesis, neuroplasticity, and the regulation of neuropeptides, as well as dysregulated cell structure and signaling (Overbey et al., 2019). Another interesting study on mouse model regards the characterization of proteomic changes in mouse brain following a 13‐day mission on the Space Shuttle Atlantis (STS‐135). The quantitative proteomic analysis showed 26 proteins altered in gray and white matter after spaceflight. These proteins were found to be associated with neuronal structure, synaptic plasticity, vesical activity, protein transport, and metabolism, suggesting the significant impact of space missions on brain structure and integrity (Mao et al., 2018).

### 4.3 Fight cancer with microgravity

Space environment represents a big challenge for the safety of human body during space missions. Microgravity may cause detrimental effects on human physiology and CNS but, of relevance, there are also a significant number of potential medical benefits related to space conditions. In the human body, cells normally grow thanks to support structures made by carbohydrates and proteins, by which they maintain their three‐dimensional shapes. On Earth, cells grow flat forming monolayers or in floating forms subjected to normal gravity. In space, cells arrange themselves into three‐dimensional aggregates, which experience reduced fluid shear stress.

Since 2014 Joshua Chou, a biomedical engineering researcher at University of Technology Sydney, started his investigations on cancer cells behavior at zero gravity, following his previous research on how to prevent bone loss in space (Bradbury et al., 2020). The study of cancer growth in altered gravity condition may have a beneficial impact on the knowledge of tumor progression mechanisms and on any potential therapeutic countermeasures.

For these reasons, increasing attentions were spent to the effects of microgravity on cellular behaviors, especially on tumor cells, as microgravity affects cell morphology, proliferation, invasion, migration, apoptosis, and gene expression. Some of the most relevant experimental evidence have been reported in Table 1 and they will be briefly discussed here. A study conducted by a genome‐wide expression profiling of colon cancer cells subjected to simulated microgravity revealed 1801 upregulated genes involved in functions associated with transcriptional regulation, proteolysis, negative regulation of cell growth, and programmed cell death, and 2542 downregulated genes involved in DNA repair, DNA replication, and cell cycle (Vidyasekar et al., 2015).

**TABLE 1:** Summary of selected articles addressing research on the effects of simulated microgravity on different cancer cells

Cell lines	Methods	Main findings	Reference
**MCF‐7** (human breast carcinoma)	Simulated microgravity with clinorotation Invasion and motility assays Immunofluorescence Gene and protein expression	Cytoskeleton disorganization Microtubule disruption Focal adhesion anomalies Decreased kinases activity (FAK, PYK2, and ILK) Cell invasion and migration retardation	Li et al. (2009)
**A549** (human lung adenocarcinoma)	Simulated microgravity with MG‐3 clinostat Wound healing assay Migration and invasion assay Gelatinolytic activity assay Proliferation assay Gene expression	Inhibition of migration, gelatinolytic activity, and cell proliferation Decrease of MKI67 and MMP2 expression Reduction of metastatic potential	Chang et al. (2013)
**MDA‐MB‐231** (human breast cancer)	Simulated microgravity with RPM Optical Microscopy Fluorescence microscopy Cell cycle analysis Annexin V assay Flow cytometry Western blot	Morphological changes Functional changes in proliferation, apoptosis, and signaling pathways (ERK, AKT, and Survivin) Cytoskeleton reorganization	Masiello et al. (2014)
**SGC‐7901** (human gastric carcinoma)	Simulated microgravity with MG‐3 clinostat Morphometric study PCNA expression Cell cycle analysis Apoptosis measurement	Alteration in cell morphology Blocking of cell conversion from the G1 to S phase Inhibition of proliferation Increased apoptosis	Zhu et al. (2014)
**DLD1** (Duke's type C colorectal adenocarcinoma) **HCT 116** (Human colorectal carcinoma) **SW620** (Human Caucasian Adenocarcinoma)	Simulated microgravity with RCCS Viability assay Apoptosis assay Gene and protein expression	Increased apoptosis Morphogenetic changes, migration, and deregulated autophagy PTEN and FOXO2 upregulation AKT downregulation CDNK2B and CDKN2D upregulation	Vidyasekar et al. (2015)
**H460** (human non‐small lung cancer cells (NSCLC)	Simulated microgravity with RPM Sphere formation assay ALDH analysis Cell cycle analysis Apoptosis analysis Gene and protein expression	Selective differentiation Increased apoptosis Stemness loss ALDH decrease Nanog and Oct‐4 downregulation	Pisanu et al. (2014)
**HCT 116** (human colorectal cancer cell line)	Simulated microgravity with RCCS Ploidy analysis Protein expression Immunocytochemistry Flow cytometry Migration assay	Increased stemness properties Increased of CD133/CD44 dual positive cells Increased autophagy Increased nuclear localization of YAP	Vidyasekar et al. (2015)
**AOS‐2**, **HOS,** and **U2OS** (human osteosarcoma) **T98G** and **U87MG** (human glioblastoma) **Du145** and **LNCAP** (human prostate adenocarcinoma) **WI38** and **H23** (human lung fibroblast and lung adenocarcinoma) **Hep3b** (human hepatocarcinoma) **Hela** (human cervical cancer) **Mewo** and **HO‐1** (human melanoma) **HN12** and **HN30** (human head and neck squamous carcinoma)	Simulated microgravity with RCCS and HFB Cell sorting Flow cytometry Annexin‐V assay Soft agar assay and sphere assay Immunofluorescence Chemosensitivity Viability assay Caspase‐3 assay	Increased CD133+ cell proliferation, especially with HFB Increased apoptosis Increased expression of CD133, CD34, CD38, Osteocalcin, Sparc, Sox‐9, RunX‐2, Stro‐1, CD117/c‐Kit, Oct3/4, Endoglin, and Integrin‐ß1 Increased chemosensitivity of CD133(+) cancer cells to various agents	Kelly et al. (2010)
**FTC‐133** (follicular thyroid carcinoma)	PFC with the Airbus A300 Zero‐G Simulated microgravity with RPM Gene array technique Quantitative real‐time PCR Cytokine measurements by MAP technology	Regulated transcription of gene involved in apoptosis, cytoskeleton, adhesion/extracellular matrix, proliferation, stress response, migration, angiogenesis, and signal transduction. Regulation of genes and proteins involved in cancer cell proliferation and metastasis, such as IL6, IL8, IL15, OPN, VEGFA, VEGFD, FGF17, MMP2, MMP3, TIMP1, PRKAA, and PRKACA Antiproliferative effects	Ma et al. (2014)
**PC‐3** (human prostatic carcinoma cell line)	Simulated microgravity with RPM Quantitative real‐time PCR Immunofluorescence Histochemical staining Time‐resolved immunofluorometric assay Pathway analysis	Multicellular spheroids growth Downregulation of the VEGF, SRC1, AKT, MTOR, and COL1A1 gene expression Upregulation of FLK1, LAMA3, COL4A5, FN1, VCL, CDH1, and NGAL Upregulations in FLT1, AKT, ERK1, ERK2, LCN2, COL1A1, TUBB, and VCL Decreased secretion of VEGFA and NGAL Cytoskeletal alterations and deposition of collagen	Hybel et al. (2020)
**U87** (human glioblastoma cells)	Simulated microgravity with 2D‐clinostat Wound healing assay Transwell invasion assay Calcium imaging Gene and protein expression	Attenuation of the invasion and migration potentials Decreased thapsigargin (TG) induced store‐operated calcium entry (SOCE) Downregulation of the expression of Orai1	Shi et al. (2015)
**C6** (rat glioma cells)	Simulated microgravity with RPM Cytoskeleton staining Scanning electron microscopy	Cytoskeleton damage Microfilaments (F‐actin) and intermediate filaments (vimentin, glial fibrillary acidic proteins (GFAP)) highly disorganized Loss of microtubules (a‐tubulin) radial array Altered chromatin condensations and DNA fragmentation	Uva et al. (2002)
**D54MG** (human glioma; wild p53) **U251MG** (human glioma; mutant p53) **T98G** (human glioma; mutant p53)	Simulated microgravity with 3D‐clinostat Proliferation assay Cell cycle analysis Chemosensitivity assay	Inhibition of growth rate Inhibition of mitochondrial activity Deceleration of mitosis Enhanced chemosensitivity to cisplatin	Takeda et al. (2009)
**U251MG** (human glioblastoma cells)	Simulated microgravity with 2D‐clinostat Cell proliferation assay TUNEL assay Human apoptosis antibody array Gene and protein expression	Inhibition of cell proliferation Induced apoptosis Upregulation of p21 Downregulation of IGFBP‐2	Zhao et al. (2017)
**HGC‐27** (gastric cancer cell line)	Simulated microgravity with RCCS Metabolic analyses by LC–MS	Identification of 67 differentially regulated metabolites Upregulation of phosphatidyl ethanolamine, phosphatidyl choline, arachidonic acid and sphinganine Downregulation of sphingomyelin, phosphatidyl serine, phosphatidic acid, L‐proline, creatine, pantothenic acid, oxidized glutathione, adenosine diphosphate, and adenosine triphosphate	Chen et al. (2020)
**FTC‐133** (follicular thyroid carcinoma)	Simulated microgravity with RPM Synthetic dexamethasone treatment (DEX) Immunofluorescence Gene and protein expression TUNEL assay Ki‐67 proliferation assay Spheroid formation assay	Suppression of spheroid formation DEX‐induced inhibition of 3D growth Regulation of Wnt/β‐catenin signaling and expression Regulation of NFKB2, VEGFA, CTGF, CAV1, BCL2	Melnik et al. (2020)
**CRL‐2351** (human breast cancer cells	Simulated microgravity with RPM Morphology evaluation Quantitative real‐time PCR	Formation of 3D spheroids Increased expression of BRCA1 Decreased expression of KRAS Upregulation of VCAM1 Downregulation of VIM	Strube et al. (2019)

Abbreviations: 2D, two‐dimensional; 3D, three‐dimensional; HFB, hydrodynamic focusing bioreactor; LC–MS, liquid chromatography‐mass spectrometry; PCR, polymerase chain reaction; PFC, parabolic flight campaign; RCCS, rotary cell culture system; RPM, random positioning machine; TUNEL, terminal deoxynucleotidyl transferase dUTP nick‐end labeling.

Real and simulated microgravity proved to induce 3D growth in many cells (Grimm et al., 2014; Ulbrich et al., 2014) by cytoskeleton reorganization and neutralization of sedimentation. Human breast cancer cells subjected to simulated weightlessness showed a loss of cytokeratin network and chromatin structure as well as an alteration of microtubules. Further, mitosis was prolonged and cell cycle blocked, with a consequent cell proliferation reduction (Vassy et al., 2003). Similarly, human MCF‐7 cells exposed to microgravity modeled by clinorotation, suffered from microfilaments and microtubules disorganization, with microfilaments which did not display the typical radial array, so that the microtubules get disrupted. Further, focal adhesions were less mature than those established in controls, with reduced number and clustering (Li et al., 2009). Microgravity simulated with clinostat has proved also to inhibit proliferation, migration, and invasion of A549 human lung adenocarcinoma cells, thus suggesting a great potential in slowing cancer metastasis (Chang et al., 2013). Also, human follicular thyroid carcinoma cell line ML‐1 showed increased amounts of apoptosis‐associated Fas protein and Bax, as well as a decrease of the antiapoptotic Bcl‐2, when cultured in simulated microgravity with clinostat (Kossmehl et al., 2003). Similarly, thyroid carcinoma cells ONCO‐DG1, gastric carcinoma cells SGC‐7901, MDA‐MB‐231 breast cancer cells, and BL6‐10 melanoma cells showed increased apoptosis under simulated microgravity (Grimm et al., 2002; Kossmehl et al., 2003; Masiello et al., 2014; Zhu et al., 2014).

This evidence confirm that compared with normal gravity, tumor cells change under altered gravity conditions, and these alterations comprise cell aggregation, cytoskeleton rearrangement, cell cycle arrest, migration, and apoptosis.

### 4.4 Effects of microgravity on CNS tumors

**FIGURE 2:**
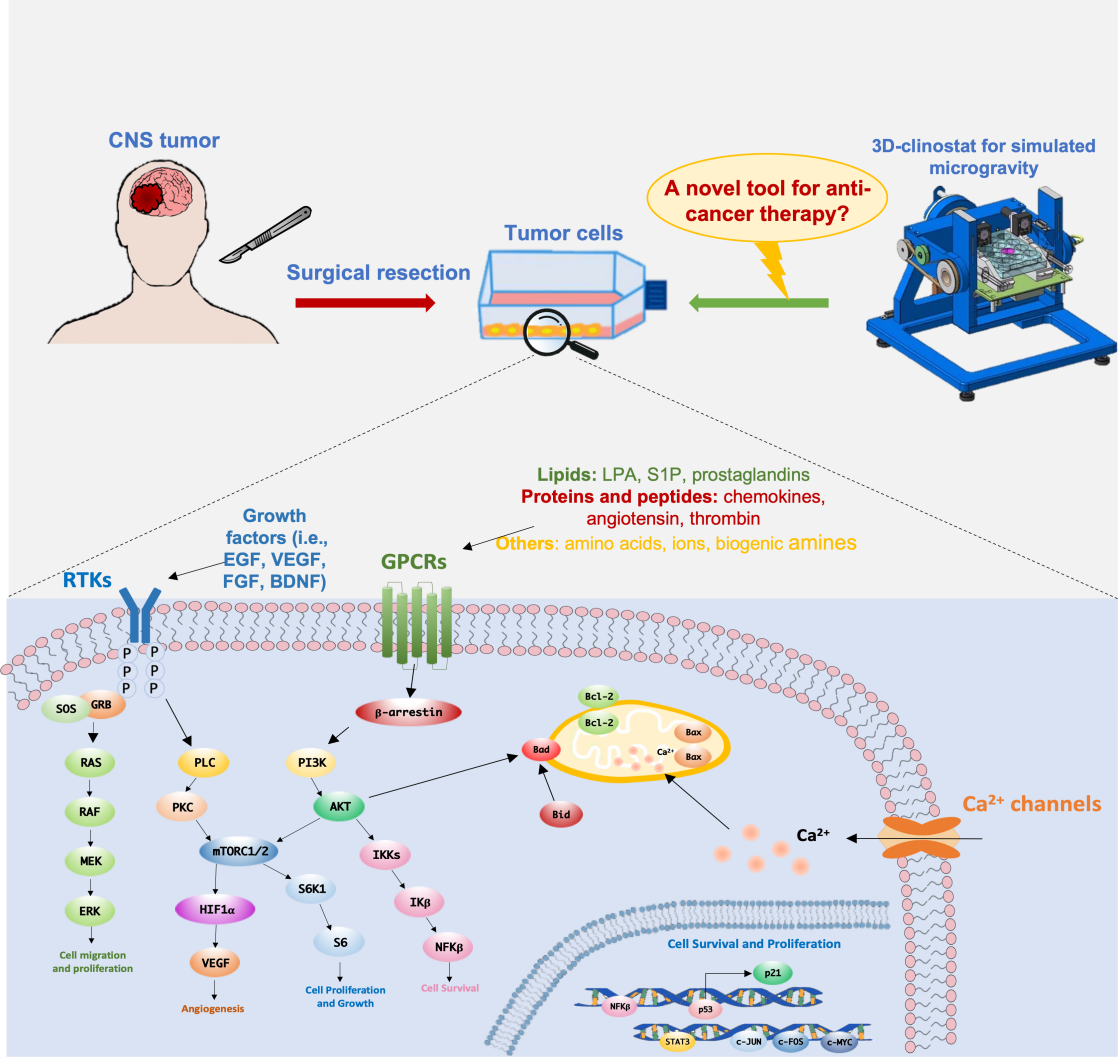
Schematic representation of malignant glioma cell signaling, in terms of cell proliferation, survival, migration, and angiogenesis. Simulated microgravity, obtainable using a 3D‐clinostat, has proven to inhibit pro‐survival cell signaling, thus promoting tumor cell apoptosis. The use of simulated microgravity to overcome tumor cell growth may be considered a novel strategy for cancer therapy, creating a promising impact on clinical practice. The image of 3D‐clinostat is derived from Borst et al. microgravity sci. Technol, 2009.

CNS tumors represent a substantial source of morbidity and mortality worldwide, affecting about 200,000 people every year and representing approximately 2% of cancer deaths (GBD 2019). The World Health Organization (WHO) specifies a grading system for CNS tumors ranging from grade I, the least aggressive with the best prognosis, to grade IV, the most malignant with worst prognosis, also known as high‐grade gliomas (HGGs). Among HGGs, glioblastoma (GBM) is the most frequent and malignant, with a poor prognosis of about 14 months and a 5‐year survival rate at 5% (Wipfler et al., 2018), representing an extreme therapeutic challenge. GBM is characterized by intense angiogenesis, invasion, cell infiltration, rapid progression, resistance to radio‐ and chemotherapies, with high frequency of relapse. The current standard protocol for GBM treatment consists in gross total resection, when possible, followed by radio‐ and chemotherapy with concomitant and adjuvant temozolomide (TMZ) (Weller et al., 2017). Despite this aggressive therapeutic regimen and the advancement of innovative strategy as molecular target therapy and immunotherapy, only weak improvements have been obtained in term of prognosis. Notably, GBM resistance to therapies and the high frequency of recurrence are mainly due to a subpopulation of tumorigenic stem‐like cells, known as GBM stem cells (GSCs), able to hierarchically initiate, maintain, and spread the neoplasm. GSCs are self‐renewing, pluripotent, highly proliferative, and genetically unstable (Pesenti et al., 2019). For these reasons, inventing novel therapeutic approaches are critically needed, and recent evidence of weightlessness in glioma cells showed a great potential for future clinical applications (Figure 2).

Several studies have been conducted using 3D‐clinostat to evaluate the effect of simulated microgravity on glioma cells. An Italian study conducted on C6‐glioma cells positioned on a 3D‐clinostat for 15 min, 30 min, 1, 20, and 32 hr reported severe alterations in cytoskeleton components after 30 min of simulated microgravity, with the disruption of the cortical layer, decrease of filopodia, microtubules appearing highly disorganized, with a loss of radial disposition. Further, the nuclear shape alteration with blebs protruded from the nuclear membrane and concurrent chromatin condensation within nuclei and DNA fragmentation were observed (Uva et al., 2002).

In another study with 3D‐clinostat, human malignant glioma cell lines D54MG, U251MG, and T98G were cultured to examine growth and chemosensitivity, compared to normal gravity condition. Results revealed that microgravity significantly decrease mitochondrial activity and cell growth rate, without impacting cell cycle. Thus, proliferation inhibition may be ascribable to mitosis slowdown. Interestingly, cells cultured in clinostat showed an enhanced chemosensitivity to cisplatin, an antitumor agent, suggesting a potential therapeutic effect of microgravity (Takeda et al., 2009). More recently, the effect of simulated microgravity has been examined on the invasion and migration ability of human glioblastoma U87 cells. The study reported that microgravity is able to attenuate the invasion and migration potentials by downregulating the expression of Orai1, a key calcium channel for store‐operated Ca^2+^ entry and decreasing the store‐operated calcium entry (SOCE). Notably, Orai1 is a novel molecular regulator of tumorigenicity and stemness, thus targeting Orai1 signaling may be a plausible therapeutic modality against cancer and microgravity might be a new good perspective in GBM therapy (Shi et al., 2015).

Another evidence come from the observation that simulated microgravity inhibits proliferation and induces apoptosis in U251 malignant glioma cells, by increasing the expression of p21 and decreasing the expression of insulin‐like growth factor binding protein‐2 (IGFBP‐2). Notably, p21 is known to suppress tumor growth by promoting cell cycle arrest, whereas IGFBP‐2 has demonstrated oncogenic functions including promoting proliferation, driving invasion, and suppressing apoptosis (Zhao et al., 2017). Also, at macroscopic level, GBM from surgery patients have been used to study the architectural alterations under the effect of simulated microgravity. To this end, GBM cells were cultured with tissue block culture method and then subjected to static culture or rotary treating for different timing. At the end of the experiment, it was observed that prolonged rotation caused an enlargement of cell somas and the degeneration of cell adhesion ability. Immunofluorescent labeling revealed a high disorganization of cytoskeleton, whit consequent microgravity‐induced apoptosis and inhibition of migration (Wang et al., 2016).

In a very recent study by Joshua Chou group, an innovative hybrid in vitro vascularized GBM‐on‐a‐chip model has been presented as a strategic integration of microfluidics and 3D bioprinting technologies, to recreate brain tumor microenvironment, comprising the functional blood–brain barrier (BBB) and the perivascular niche. This model was used to evaluate the effect of microgravity condition, in terms of mechanobiology, representing a novel and helpful tool for preclinical approach in neuro‐oncology. Using the RPM, it would be possible to observe that the absence of gravity inhibits GBM cell invasion and aggregation, without affecting cell viability. This in turn confirm that GBM spheroid formation, typical of GSC growth is gravity dependent, so that microgravity compromises GBM basic cell functions and mechanisms (Silvani et al., 2021).

In 2009, the GlioSat/GlioLab project started to study the combined effects of microgravity and ionizing radiation on survival rate of GBM cancer cells. By this project, promoted by GAUSS (Group of Astrodynamics of University of Roma La Sapienza) at the School of Aerospace Engineering in Roma, the Space Science Center in Morehead State University in Kentucky, and the Genetic department of IRCCS‐Hospital CSS San Giovanni Rotondo in Italy, a cell set was monitored in parallel in orbit and on the ground, to compare tumor cell behavior. To date, two preliminary flights on board of the Space Shuttle have already been done, STS‐134 and STS‐135, and the results obtained on the DNA analysis give a confirmation of the original idea of GLioLab/Gliosat project, justifying the pursuance of the investigation (Cappelletti et al., 2012).

An innovative series of research investigations is making further advancements in cancer therapy using the peculiar microgravity space environment aboard the ISS. In particular, a process known as microencapsulation is able to produce tiny, liquid‐filled, biodegradable micro‐balloons containing specific combinations of antitumor drugs, which may be delivered directly to specific sites within a cancer patient, effectively revolutionizing cancer treatment. The microencapsulation experiments have been conducted by Dr. Dennis Morrison of NASA's Johnson Space Center, aboard the space station as a tool to develop the Earth‐based technology, called the Microencapsulation Electrostatic Processing System‐II (MEPS‐II). In laboratory tests, MEPS‐II microcapsules carrying antitumor drugs have been injected into human lung and prostate tumors in animal models, demonstrating improved site‐specific therapeutic results and the inhibition of tumor growth (https://www.nasa.gov).

A few years ago, Angiex, a biotechnology company based in Cambridge, Massachusetts, settled a Model System for Evaluation of Cancer Therapy Toxicity, examining the proliferation of endothelial cells (ECs) that constitute blood vessels, in microgravity condition. The hypothesis of the study is that microgravity‐cultured ECs represent a valid model to test the effects of vascular‐targeted agents. The study may facilitate a cost‐effective method that does not require animal testing, and which may help develop safer and more effective vascular‐targeted drugs (https://www.nasa.gov).

Regarding in vivo cancer models, most of the studies have been focused on immune system as microgravity may cause changes in both innate and adaptive immune systems.

For example, during Soyuz, Skylab, Salyut, and Space Shuttle programs, a reduction in immune cell function was observed to be directly dependent to flight times, with a 50% decrease in lymphocytic response after mission compared to the preflight (Konstantinova et al., 1993; Topal & Zamur, 2021). Among the effects of microgravity on immune cells in mice, it has been reported a suppression of lymph cell mitogenesis and cytotoxic T cell activity, together with a reduction of T cell percentages, numbers, response to a strong mitogen, and secretion of cytokines, which are critical mechanism for an optimal immune defense and homeostasis (Gridley et al., 2009; Sonnenfeld et al., 1992). A study by Shi et al. using mouse hematopoietic stem and progenitor cells reported under simulated microgravity and reported that microgravity had a significant reduction effect on macrophage number, differentiation, and functional polarization, with consequent changes in gene expression profiles (Shi et al., 2021).

Immune system and cancer have a close relationship, as immune response disorders may contribute to cancer onset or resistance to therapy. Several studies demonstrated the association between microgravity and immune system, comprising immune cell activation and inflammatory cytokine production (Corthay, 2014; Mukhopadhyay et al., 2016). Therefore, we can assert that microgravity may represent a promising strategy for immunomodulation.

Taken together, experimental studies conducted on space environment exposure really enhance our knowledge on the role of microgravity in cancer cell growth, serving as novel paradigm for innovative strategies. The combination of the available resources in the characteristic space environment with the advanced Earth technologies may allow us to better understand the pathologies and to study and experience countermeasures as well for the future space missions as for the terrestrial diseases.

## 5 CONCLUSION

Space presents an unlimited horizon for investigation and discovery. A wide range of molecular, physiological, and morphological changes occur in cells and tissues exposed to altered gravity conditions. The microgravity‐induced alterations in physiological cell biology, as cell cycle progression, cytoskeleton remodeling, and apoptotic mechanisms are straight translable in the medical field, especially neuroscience and oncology, accelerating and promoting life science research. Of relevance, the comprehension of the effects of microgravity on CNS functions and the understanding of the basic mechanisms by which these effects occur will be of direct benefit to optimize the impact of and providing countermeasures for log‐term exposure of humans to the weightlessness of spaceflight and partial gravity. In this context, it would be really stimulating to create a multidisciplinary taskforce to conduct, merge, and optimize different topic studies and create a precise picture of functional, structural, and biochemical brain alterations associated with spaceflight.

## CONFLICT OF INTEREST

The authors declare no conflict of interest.

## AUTHOR CONTRIBUTIONS

GM, SEN, LG, and EG contributed to conception, investigation, writing‐original draft and writing‐review and editing. RC, ML, MM, PP, GDM, LC, PT, CC, ML, GCA, and AL contributed to investigation, visualization and writing‐review and editing. All authors read and approved the final manuscript.
